# Long‐Term Visual Gist Abstraction Independent of Post‐Encoding Sleep

**DOI:** 10.1111/jsr.70106

**Published:** 2025-06-09

**Authors:** Nicolas D. Lutz, Johanna Himbert, Jessica Palmieri, Eva‐Maria Kurz, Isabel Raposo, Xuefeng Yang, Jan Born, Karsten Rauss

**Affiliations:** ^1^ Institute of Medical Psychology and Behavioral Neurobiology University of Tübingen Tübingen Germany; ^2^ Institute of Medical Psychology, Faculty of Medicine LMU Munich Munich Germany; ^3^ Department of Child and Adolescent Psychiatry, Psychosomatics, and Psychotherapy University Hospital of Psychiatry and Psychotherapy Tübingen Germany; ^4^ Graduate Training Centre of Neuroscience/IMPRS for Cognitive and Systems Neuroscience University of Tübingen Tübingen Germany; ^5^ Werner Reichardt Centre for Integrative Neuroscience University of Tübingen Tübingen Germany; ^6^ German Center for Diabetes Research (DZD), Institute for Diabetes Research and Metabolic Diseases of the Helmholtz Center Munich at the University of Tübingen (IDM) Tübingen Germany; ^7^ German Center for Mental Health (DZPG) Tübingen Tübingen Germany

**Keywords:** abstraction, consolidation, gist, memory, sleep

## Abstract

Current theories of memory processing postulate a slow transformation from episodic to abstract, gist‐like memories. We previously demonstrated that sleep shortly after learning improves gist abstraction in healthy volunteers across a one‐year retention interval using a visual version of the Deese‐Roediger‐McDermott (DRM) paradigm. Here, we investigate the temporal evolution of this effect by testing recognition performance on a similar DRM task immediately after encoding, as well as 1 week and 1 year later. Moreover, we address the role of feature overlap during encoding, using stimulus sets that are either closely related to or more distant from their common prototype. Behavioural data were obtained from *N* = 16 healthy volunteers in a within‐subjects design, where different sets of shapes were learned in separate experimental sessions, followed by consolidation during day‐time wakefulness or nocturnal sleep, respectively. Our results indicate high levels of (false) recognition of non‐encoded prototypes for all measurement points, including after 1 year. However, in contrast to our previous findings, gist memory was not affected by whether participants slept or stayed awake during the first 12 h after encoding. Comparisons across experiments indicate that the divergent results are due to changes in task demands rendering item and gist memory traces less distinct in the present study. Our results confirm the behavioural persistence of visual gist abstraction across extended intervals. At the same time, they highlight that sleep effects on this process are highly dependent on task demands.

## 1 Introduction

The benefits of sleep for memory consolidation were originally demonstrated using unrelated stimuli such as individual syllables (Jenkins and Dallenbach 1924) or words (Gais et al. 2006). However, research over several decades has stimulated the idea that sleep is at least as important for extracting and consolidating higher‐order structure contained in newly encoded information (Brodt et al. 2023; Landmann et al. 2014; Lerner and Gluck 2019; Lewis and Durrant 2011; Wagner et al. 2004). Accordingly, current theories of memory processing postulate a slow transformation from episodic to more abstract memories, with the latter based on generalised representations that are often referred to as the ‘gist’ of the encoded information (Dudai 2012; Winocur and Moscovitch 2011; Yassa and Reagh 2013). Gist‐based recognition and reasoning are thought to support adaptive behaviour by improving generalisation to novel instances while retaining a reliable record of central stimulus features (Zeithamova and Bowman 2020).

However, gist abstraction may also impair memory performance (Tompary and Davachi 2017). Indeed, one of the most prominent areas of research into gist abstraction are studies investigating ‘false’ memories: in the classic Deese‐Roediger‐McDermott (DRM) paradigm (Deese 1959; Roediger and McDermott 1995), participants learn lists of words that are highly semantically related (e.g., ‘bed’, ‘dream’, ‘night’, ‘rest’). At a later retrieval test, participants often remember the gist of the learned lists (e.g., ‘sleep’), and they usually do so with high subjective confidence, despite not having seen these lures during learning. It has been argued that such findings indicate gist abstraction, since the non‐veridical recognition of the common denominator of multiple stimuli is likely useful in real‐world settings (Cann et al. 2011; Diekelmann et al. 2010; Kurz et al. 2019; Kurz et al. 2021; Pardilla‐Delgado and Payne 2016; Payne et al. 2009). However, this conclusion is questionable given that performance in the DRM paradigm may reflect the characteristics of pre‐existing semantic networks rather than abstractions. Nevertheless, similar results have been reported when abstract visual stimuli are used as learning material (Homa and Hibbs 1978; Posner and Keele 1968, 1970; Zeng et al. 2021). Here, in the absence of high‐level knowledge about the stimulus domain, ‘false’ memories are more likely to reflect active processes of abstraction.

Gist abstraction is thought to benefit from sleep (Dudai et al. 2015; Inostroza and Born 2013; Lewis and Durrant 2011). However, the picture emerging from studies that tested the effects of sleep on gist abstraction is inconclusive (Newbury and Monaghan 2019): whereas some studies showed an enhancing effect of sleep (Diekelmann et al. 2010; McKeon et al. 2012; Pardilla‐Delgado and Payne 2016; Payne et al. 2009), others found no difference or even a reduction of gist knowledge after sleep compared with wakefulness (Darsaud et al. 2011; Fenn et al. 2009; Lo, Sim, et al. 2014).

One factor that may have contributed to the mixed outcomes of previous studies is the delay of testing, which in most of these studies was relatively short. Earlier studies have suggested that gist abstraction is a process that evolves slowly over time (McDermott 1996; Neuschatz et al. 2001; Payne et al. 1996; Posner and Keele 1970; Seamon et al. 2002; Strange et al. 1970; Thapar and McDermott 2001; Toglia et al. 1999). In a previous study (Lutz et al. 2017), we used a non‐verbal, visual version of the DRM paradigm to test this idea. We found a beneficial effect of sleep on gist abstraction only after a retention interval of 1 year, while recall of veridical memories was improved immediately after sleep vs. wakefulness (Lutz et al. 2017). Moreover, we observed high recognition rates for gist‐like prototypes in a baseline test early after encoding. These findings suggested that abstraction may occur early after encoding, but then requires sleep to be retained for the long term.

A second open question is whether the temporal evolution of gist abstraction depends on the extent to which features of different stimuli overlap (Bowman and Zeithamova 2020). It has been shown that sleep is particularly involved in consolidating memory traces of weak‐to‐intermediate strength (Denis et al. 2021; Drosopoulos et al. 2007; Lutz et al. 2024; Stickgold 2009). This suggests that encoding strength would affect both the degree to which sleep promotes abstraction, as well as its temporal evolution. However, to our knowledge, this has not been systematically investigated.

In the present study, we address the nature and dynamics of visual gist abstraction using an adapted version of our previously established protocol (Lutz et al. 2017). We had participants learn stimuli of different degrees of similarity with their respective prototypes and measured abstraction immediately after encoding, after 1 week, and after 1 year. We hypothesized that sleep (vs. wakefulness) improves gist abstraction (i.e., higher recognition for prototypes vs. encoded stimuli) 1 week after encoding (compared with an immediate test), particularly for stimuli that share little (vs. more) feature overlap. We further hypothesized that this beneficial effect of sleep on gist abstraction is maintained over longer periods of time and would still be visible 1 year after encoding.

Our results confirm that gist memory persists for up to 1 year, but we did not observe a beneficial effect of sleep on visual gist abstraction at either time point. To better understand the reasons for these equivocal findings, we compared the present data to our previous experiments and found that changes in task parameters led to differences in baseline performance which likely reduced benefits of sleep for gist memory abstraction and consolidation.

## 2 Methods

### 2.1 Participants

Based on the parameters and results of our previous study (Lutz et al. 2017), 16 healthy participants (8 female, 8 male; mean age: 23 years; range: 18–33) took part in the present experiment. Two participants were excluded from analyses of sleep parameters due to technical issues (for one participant, no EEG was recorded) or poor EEG quality (non‐stereotypic artefacts, potentially related to electrodes becoming detached overnight). Furthermore, only 12 of the original 16 participants were available for retesting after 1 year. Participants were not on any medication, caffeine, or alcohol, and did not suffer from any neurological or psychological disorder. In addition, participants had a normal sleep–wake cycle (i.e., their usual bedtime was between 2200 and 2400 h and they usually got up between 0700 and 0900 h), had a normal or corrected‐to‐normal visual acuity, and were not colour‐blind. All participants gave written informed consent and were paid for participation. The experiment was approved by the ethics committee of the Medical Faculty at the University of Tübingen and conducted in accordance with the approved guidelines.

### 2.2 Stimuli and Task

**FIGURE 1:**
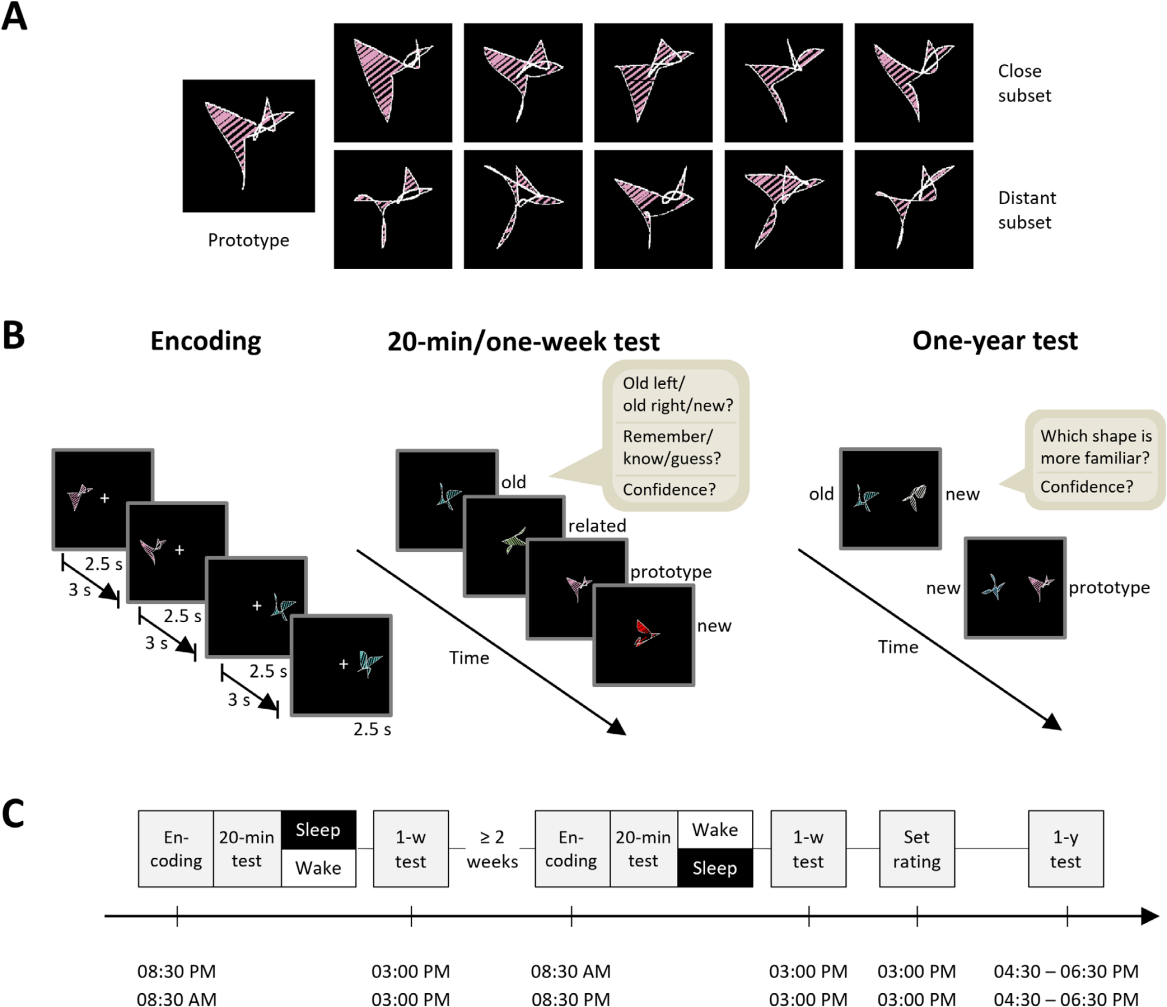
(A) Example stimulus set. Stimulus sets consisted of 10 abstract shapes derived from a single prototype. Every set was split into two subsets containing either the five shapes rated most similar to the prototype (“close” subset), or the five shapes rated least similar to the prototype (“distant” subset) in a pilot study on a separate sample of participants (*N* = 14). (B) Memory task. In each experimental condition (see below), participants encoded 40 sets of 5 abstract shapes (either close to or distant from the prototype; repeated twice in a row), presented either on the left or right side of a computer screen (only two shapes per set shown for simplicity). At the test after 20 min or 1 week, participants were presented with studied old shapes, non‐studied related shapes from the same set, non‐studied prototype shapes, as well as new shapes. Memory performance for old shapes was taken as an index of veridical memory, whereas false recognition of prototypes was used to operationalize gist memory. False recognition of related shapes was used to test whether prototypes were indeed perceived as prototypical (see Methods and Supplementary Information for details). For each shape, participants were asked if it is old (i.e., if they have seen it before) or new. If they thought it was old, they should also indicate whether it was presented on the left or right side of the screen. Participants should further indicate whether they remember, know, or guess and how confident they are about their choice (4‐point scale). Finally, during the one‐year test, participants performed a two‐alternative forced‐choice (2‐AFC) task, in which they were presented with either an old shape or prototype on one side of the screen and a new shape on the other side of the screen, and they had to decide which of them is more familiar. Afterwards, they were asked to indicate their confidence about their choice on a 4‐point scale. (C) Experimental design. Each participant was tested in a Sleep and a Wake condition. In the Sleep condition, participants encoded the stimulus material in the evening, and in the Wake condition, they encoded the stimulus material in the morning. Twenty minutes afterwards, there was a first test (20‐min test), followed by a test after a one‐week delay (one‐week test). At the end of participants' two experimental conditions, they took part in a set rating. Approximately 1 year later, participants were tested again (one‐year test). Testing was done in counterbalanced order between the two conditions. Different sets of stimuli were used for the two conditions and the 20‐min and one‐week tests. During the one‐year test, stimuli from both conditions were compared with new stimuli.

To test the sleep‐dependent evolution of gist abstraction, we employed an adapted version of the non‐verbal visual DRM paradigm (Diekelmann et al. 2011; Lutz et al. 2017; Slotnick and Schacter 2004) and tested participants across different time intervals (20 min after encoding, after a delay of 1 week, as well as after ~1 year of retention; see *Design and procedure*). The total of 80 employed stimulus sets each consisted of 10 abstract exemplar shapes derived from a single prototype (Figure 1A) (Slotnick and Schacter 2004). For the present study, every set was split into two subsets based on the results of a pilot experiment in a separate sample of participants (*N* = 14): subsets either contained the five shapes rated most similar to the prototype (“close” subset) or the five shapes rated least similar to the prototype (“distant” subset). On average, participants rated the subsets' distance from their prototype at a median of 3 and 8 on a scale of 1 to 10 (with 1 being closest and 10 being most distant). These ratings were significantly different for all sets individually (Wilcoxon signed‐rank test, *p* ≤ 0.011) except for two (out of 80), which showed a statistical trend (*p* ≤ 0.088).

During encoding, the shapes of a given subset were presented on the screen one after another, twice in a row in the same order. Presentation time was 2.5 s per shape, with an inter‐stimulus interval of 3 s (Figure 1B, left). In each experimental condition (sleep/wake), participants were shown shapes from 40 stimulus sets, with half of the sets presented as close subsets and the other half as distant subsets. Participants were informed about the repeated presentation of shapes from the same set. Stimuli appeared at one of two pre‐defined locations on the screen (left or right), with all stimuli of one set presented at the same location. The participants' task was to memorise the visual features and location of every single shape. Presentation of stimuli followed specific rules to ensure comparability between conditions: (1) presentation of close or distant shapes of a set was counterbalanced across participants and conditions. (2) To ensure comparable variances of stimulus sets across conditions, sets of comparable perceived similarity were sampled and counterbalanced across conditions. Stimulus sets were ranked according to the perceived similarity of their items with the respective prototype, with similarity judgements obtained in a pilot study on a separate sample (*N* = 14). Similarity judgements were transformed into a measure of “internal consistency”, and the continuum of internal consistencies was split into eight bins of equal width. From each of these bins, five sets were sampled per condition, resulting in 40 sets containing 200 individual stimuli per condition. (3) Presentation on the left and right side of the screen switched after each set, with the starting condition counterbalanced across participants and presentation locations counterbalanced across conditions for each set. (4) No more than three close or distant subsets were presented in a row. In addition, the order of the sets was randomly selected for each participant.

During the 20‐min and one‐week tests, participants were presented with 20 sets each, that is, half of the encoded sets were tested during the 20‐min test and the other half was tested during the one‐week test. Four types of shapes per set were presented: one ‘old shape’ from the encoded subset; one ‘related shape’ from the corresponding unseen subset of the same set; the non‐studied ‘prototype’ of each set; and three ‘new shapes’ from non‐studied sets (with each triplet taken from the same set; Figure 1B, middle). To minimise variance in similarity between prototypes and old or related shapes, we only presented participants with shapes that were previously rated to be of intermediate distance in the close and distant subsets (i.e., we always presented the third out of 5 shapes in each subset, after sorting them from closest to most distant on the basis of rating data from an independent sample as described above). Unlike during encoding, in this recognition test, shapes were presented in the middle of the screen. For each shape, participants had to indicate if they had seen it before and where it was presented (i.e., they were presented with the choices “old‐left”, “old‐right” and “new”). As there was no effect of position recall between conditions (*p* ≥ 0.198 for main and interaction effects with Sleep/wake), data were collapsed across this measure. Afterwards, they were asked to make remember/know/guess judgements and indicate their confidence on a 4‐point scale. To reduce potential recognition biases due to shapes of different sets being presented with the same sequential pattern, we pseudo‐randomly selected the first stimulus from each encoded set seen in a test session to be either an old shape, a related shape, or a prototype shape with equal probability of 1/3. It was also ensured that the same stimulus type from different sets (old, related or prototype shapes) was not presented more than twice in a row. Apart from these rules, presentation of stimuli was again randomised for every participant. The recognition test was followed by an exploratory familiarity rating where the same stimuli that were presented during the recognition test (one prototype, one old shape and one related shape per set) were simultaneously shown on the screen. Participants’ task was to rank them according to their familiarity (from most to least familiar). Data for this task are not shown due to the exploratory nature of this task and largely inconclusive results.

In a separate session following the one‐week test, participants took part in a set rating task. To determine if the prototypes really represent the gist of their respective sets, participants were presented with all 11 shapes (i.e., the 10 exemplars plus the prototype) of each set. They were then asked to pick two shapes they thought represented the whole set best, with the most representative shape to be selected first. All 80 sets (i.e., 40 sets in each condition) were rated. The location of the shapes was randomised for each participant.

Finally, at the one‐year test, participants were simultaneously presented with two stimuli (either an old shape vs. a new shape or a prototype vs. a new shape; Figure 1B, right). In this two‐alternative forced‐choice (2‐AFC) task, for each pair, participants were asked which of the two shapes was more familiar to them. Additionally, they were asked for their confidence on a 4‐point scale. As in the 20‐min and one‐week tests, we used one of the old shapes (that had been seen three times, twice during encoding and once during set rating, but had not been tested during recognition) and the prototype (which had also been seen three times, during recognition test, familiarity rating, and set rating) of each of the 80 sets. This was balanced by an equal number of new shapes. We used two shapes from each new set (thus matching the number of shapes per encoded set, one old shape and one prototype), that is, the 160 new shapes were derived from 80 unseen sets. Presentation of stimulus types was balanced across the two locations. Furthermore, as in the 20‐min and 1‐week tests, the shapes were pseudo‐randomly selected such that for one half of the sets, the first stimulus presented during the test was an old shape and for the other half of the sets, it was the prototype shape. In addition, at least three shapes from other sets were shown before another shape of the same set was shown again.

### 2.3 Design and Procedure

Participants took part in two conditions in a within‐subjects design: in the Sleep condition, they were initially tested 20 min after encoding before a night of normal sleep; in the Wake condition, the same participants were initially tested 20 min after encoding before normal daytime wakefulness. Subsequent tests took place after a delay of 1 week, as well as after ~1 year of retention (Figure 1C).

Half of the participants started with the Sleep condition; the other half started with the Wake condition. In addition to the experimental sessions, 2–3 days before the Sleep condition, there was an adaptation night to familiarise participants with sleeping in the sleep lab environment.

Inclusion and exclusion criteria were assessed using simple questionnaires, as well as the Freiburg Vision Test, FrACT (Bach 2007), and a colour vision online test (http://www.color‐blindness.com/color‐arrangement‐test/) when participants first arrived at the sleep lab. Additionally, in the adaptation night session, participants took part in a word fluency test (Aschenbrenner et al. 2000) and a handedness test (Oldfield 1971). During the adaptation night, participants had the opportunity to sleep in the laboratory with the electrode setup for polysomnographic EEG recordings between 11:00 PM and 7:00 AM. The next morning, they completed a questionnaire on sleep quality (the German “Schlaffragebogen A/Revidierte Fassung” [SF‐A/R]; Görtelmeyer 2011) and left the laboratory.

On the day of the encoding session, participants came to the laboratory between 8:00 PM and 08:15 PM (Sleep condition) or 8:00 AM and 8:15 AM (Wake condition). After initial tests investigating participants' vigilance (Diekelmann et al. 2013) and subjective sleepiness (Stanford Sleepiness Scale; Hoddes et al. 1972), encoding of the stimulus material started at 8:30 PM (Sleep condition) or 8:30 AM (Wake condition). Participants were again tested on their sleepiness at the end of encoding, and then performed a first test after 20 min of relaxing music (20‐min test; Lutz et al. 2017).

This was followed by a first debriefing (asking participants how difficult they found it to encode the stimulus material, if they used a particular encoding technique, and how difficult it was for them to recognise the shapes in the test), and a digit span test. Afterwards, in the Sleep condition, participants had the possibility to sleep in the sleep laboratory for 8 h, from 11:00 PM until 7:00 AM, including polysomnographic recordings. The next morning, they completed another questionnaire on sleep quality (SF‐A/R) and left the laboratory. In the Wake condition, participants left the laboratory after the digit span test. Participants were instructed to keep a regular sleep–wake cycle and refrain from taking day‐time naps during the retention intervals. For this purpose, their motor activity was controlled by actimetry (MotionWatch 8, CamNtech Ltd., Cambridge, UK) in both conditions. Additionally, we used a questionnaire to record participants' daily activities. Participants also filled a brief sleep diary until they returned for the one‐week test.

The one‐week test session took place 7 days after encoding at 3:00 PM. The test was preceded by initial tests on vigilance and sleepiness, and followed by another debriefing (asking how difficult participants found it to recognise the stimulus material, and, if this was their second condition, if they noticed similarities between stimuli within or across sets), a digit span test, a test on their general sleep quality (Pittsburgh Sleep Quality Index, PSQI) and chronotype (Morningness‐Eveningness Questionnaire, MEQ; Horne and Ostberg 1976). Participation in the two conditions was separated by an interval of at least 2 weeks. All participants were invited for an additional set rating session after finishing both conditions. The set rating took place at 3:00 PM and was again preceded by tests on vigilance and sleepiness.

Finally, 12 participants of the original sample took part in a one‐year test (mean delay from initial encoding, 368 ± 26 days; cf. Lutz et al. 2017). All participants were tested between 4:30 and 6:30 PM. Before this final test, we again tested participants' vigilance and sleepiness.

### 2.4 Data Acquisition and Analysis

Behavioural data was acquired using MATLAB/Psychtoolbox (RRID:SCR_002881; Brainard 1997; Kleiner et al. 2007; Pelli 1997). We measured accuracy of task performance and reaction times. Gist memory was assessed in terms of recognition performance for the prototype shapes; veridical memory was assessed as the recognition performance for old shapes. For correlational analyses, we additionally calculated a difference score between recognition of prototypes and old shapes (prototypes minus old shapes). Finally, the recognition performance (percentage of “old” responses) for shapes of the non‐studied half of a set (here referred to as “related” memory) was contrasted with prototypes as a measure of prototypicality (i.e., to investigate if prototypes were indeed perceived as more prototypical, given that both prototypes and related shapes were not seen during initial encoding; see Figure S1).

To assess whether and how participants slept during the one‐week interval, we collected actigraphy data and used additional questionnaires. Participants were asked to press a marker on the actigraph (MotionWatch 8) when switching off the lights in the evening and getting up in the morning. In the subjective questionnaires, participants should indicate the time they switched off the lights, the time when they fell asleep, how often and how long they were awake during the night, as well as the time when they got up. Time in bed (TiB) was calculated as the time between lights off and getting up; sleep period was calculated as the time between falling asleep and waking up; sleep onset latency (SOL) was calculated as the difference between lights off and the time falling asleep; and wake after sleep onset (WASO) was calculated as the time being awake after falling asleep. For the analysis of the actigraphy data, we used a standard algorithm as implemented in MotionWare 1.2.5 (RRID:SCR_022253), with a high sensitivity threshold for the detection of wake phases and an epoch length of 15 s.

Polysomnographic recordings were used to monitor brain activity in the Sleep condition during the experimental night in the laboratory. The electroencephalogram (EEG) was recorded at eight locations (F3 and F4, C3 and C4, P3 and P4, and O1 and O2, according to the international 10–20 system). The electrodes were referenced online against the mean of the two mastoids (A1, A2). Additionally, the electrooculogram (EOG) and electromyogram (EMG, electrodes positioned on the chin) were recorded with bipolar montages. Sleep scoring was performed manually following standard criteria (Rechtschaffen and Kales 1968). Offline analyses of slow oscillations (SO) and sleep spindles were performed using algorithms implemented in SpiSOP (RRID:SCR_015673), based on sleep stages S2, S3, and S4 and using standard parameters. Spindles were detected in a range of ±1 Hz around the individual frequency peak (mean = 13.22, SD = 0.5) in the spindle frequency range based on channel‐averaged power spectra. Given the visuo‐perceptual nature of our task, our focus was on occipital SOs. Thus, SO measures were averaged across electrodes O1 and O2. Co‐occurrence of spindles and SOs was assessed in two ways. First, within each participant and each electrode site, the percentage of spindles coinciding with an SO (SO trough) ± 1.2 s around the spindle trough was determined and then averaged across all electrode sites. Secondly, only for spindles coinciding with an SO between the two positive‐to‐negative zero crossings of an SO (Denis et al. 2021; E.‐M. Kurz et al. 2023), the signal (±3 s around SO trough) was filtered using a 0.3 Hz high‐pass filter and a 3.5 Hz low‐pass filter. The same signal was then filtered between ±1 Hz around the individually determined spindle frequency peak. The Hilbert transform was then applied to each time series to extract the instantaneous phase of the SO filtered signal and the instantaneous amplitude of the spindle frequency filtered signal, respectively. For each SO‐spindle event, the SO phase at the spindle amplitude maximum was then extracted and averaged across electrodes within a participant using the circstat toolbox (Berens 2009) in MATLAB.

### 2.5 Statistics

Two‐tailed tests were chosen for all statistical analyses. The level of significance was set to *p* < 0.05. Repeated‐measures ANOVAs as implemented in JASP (JASP Team 2024) were used, in combination with follow‐up *t*‐tests. Greenhouse–Geisser correction of degrees of freedom was applied when the assumption of sphericity was violated. We report original degrees of freedom and corrected *p* values in these cases.

## 3 Results

We tested the temporal evolution of sleep‐associated visual gist abstraction and the role of stimulus similarity in this process. Participants encoded sets of abstract shapes and performed a first individual‐stimulus recognition test of half of the encoded stimulus sets after 20 min. Performance for prototypical shapes not seen during encoding was used as an index of gist abstraction. This was followed by either nighttime sleep or daytime wakefulness in separate experimental sessions, with normal sleep–wake patterns indicated by polysomnography (PSG) recordings (Table S1) and actigraphy measurements (Table S2). A second recognition test was performed 1 week later for the other half of the encoded sets, thus ensuring that prototypical stimuli used to assess gist memory were not seen by the participants before recognition testing during either the 20‐min or the one‐week test. Subsequent control tests confirmed that prototype shapes were indeed perceived as prototypical (Figures S1 and S2; see also Figure S3). Finally, participants returned to the laboratory for a 2‐AFC recognition task after a delay of one year.

### 3.1 Sleep Does Not Improve Gist Abstraction as a Function of Feature Overlap

**FIGURE 2:**
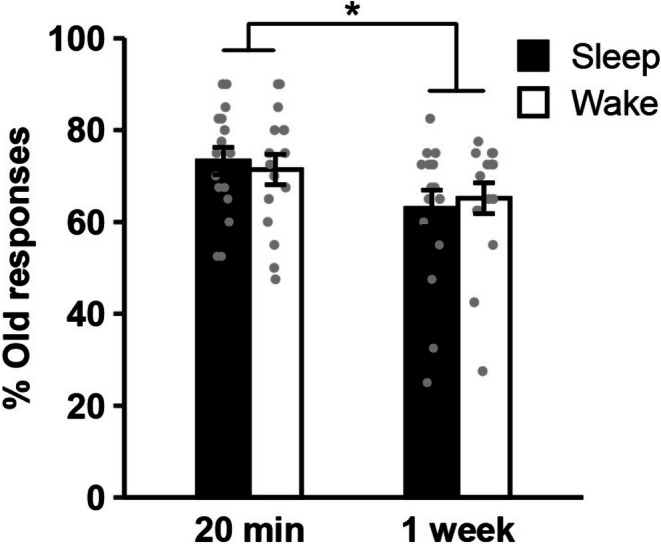
Overall memory performance after 20 min and 1 week. Means ± SEM and individual data points are shown for overall performance (% old responses across prototypes and old shapes), separated for Sleep and Wake conditions in the 20‐min and one‐week tests. *p < 0.05 for the interaction between Sleep/Wake and 20‐min/1‐week. *N* = 16.

To test whether the evolution of gist memory across the first week after encoding is modulated by stimulus similarity and sleep immediately after encoding, we conducted a repeated measures ANOVA with factors Sleep/Wake condition, 20‐min/1‐week tests, Prototype/Old stimuli and Close/Distant stimulus sets. The results indicated globally lower recognition rates after 1 week (main effect 20‐min/1‐week, *F*(1, 15) = 5.38, $\eta_{p}^{2}=0.26$, *p* = 0.035). This effect was driven by *lower* performance if participants slept immediately after encoding (20‐min/1‐week × Sleep/Wake, *F*(1, 15) = 5.22, $\eta_{p}^{2}=0.26$, *p* = 0.037; Figure 2). No other main or interaction effects linked to sleep were significant (remaining *p* ≥ 0.272). In particular, the hypothesized four‐way interaction of all factors was far from significant (F < 1). Also, encoding close or distant stimulus subsets did not change recognition performance in any condition (all *p* ≥ 0.148). In addition, confidence ratings and remember/know/guess judgements did not indicate any differences between Sleep and Wake conditions (all *p* ≥ 0.125; Table S3). Additional control tests on vigilance, sleepiness, and digit span further did not differ between Sleep and Wake conditions at any point of time (Table S4).

**FIGURE 3:**
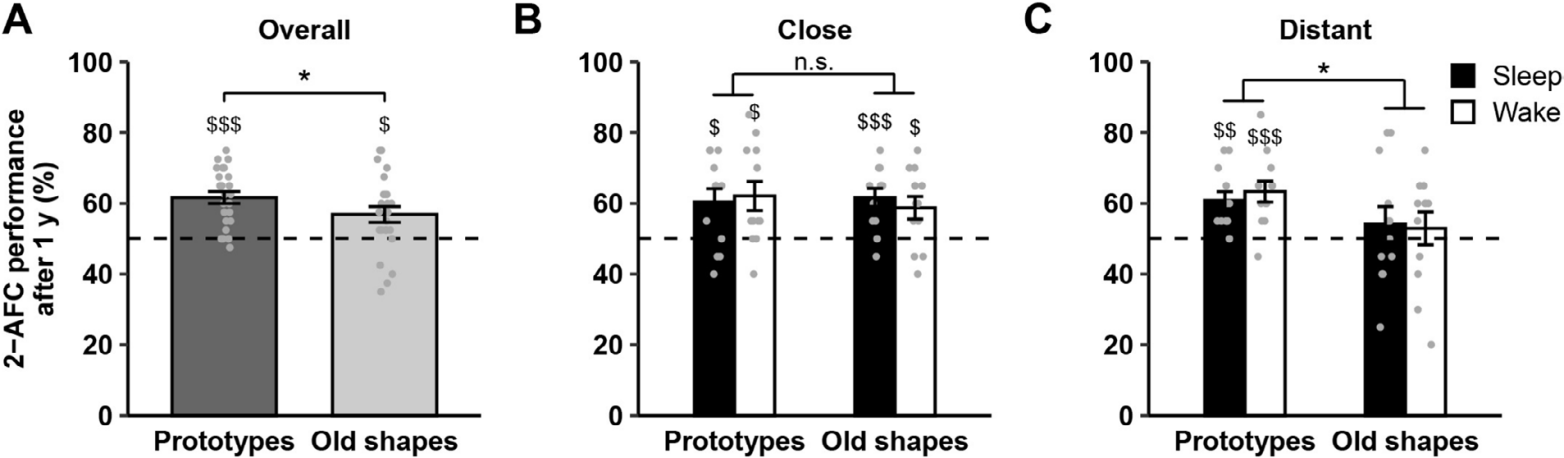
One‐year test. Means ± SEM and individual data points for prototypes and old shapes are shown for 2‐AFC performance at the one‐year test. (A) Overall performance across Sleep and Wake conditions. (B) Performance for stimulus sets similar to the set prototype (close sets), split for Sleep and Wake conditions. (C) Performance for distant sets, split for Sleep and Wake conditions. A marginally significant interaction between stimulus type and stimulus similarity (Close/Distant × Prototype/Old, *p* = 0.079) was followed up with post hoc indicating preferential consolidation of prototype information for distant stimulus sets. *p < 0.05 for comparisons between stimulus types; ^$$$^ *p* < 0.001, ^$$^ *p* < 0.01, ^$^ *p* < 0.05 for comparisons against chance level (50%); n.s., not significant. *N* = 12.

To test whether gist memory persists across extended intervals, we invited participants back to the laboratory after ~1 year. In a 2‐AFC task, they selected the more familiar of two stimuli presented simultaneously: either an old shape vs. a new shape or a prototype vs. a new shape. A repeated‐measures ANOVA with factors Sleep/Wake condition, Prototype/Old stimuli and Close/Distant stimulus sets showed better performance for prototypes compared with old shapes (main effect Prototype/Old, *F*(1, 11) = 5.45, $\eta_{p}^{2}$ = 0.33, *p* = 0.039; Figure 3A). This effect was marginally more pronounced for stimuli from distant subsets (interaction Prototype/Old × Close/Distant, *F*(1, 11) = 3.76, $\eta_{p}^{2}$ = 0.26, *p* = 0.079; post‐hoc tests: Distant, *t*(11) = 2.54, Cohen's *d* = 0.67 [95% CI: −0.29, 1.63], *p* = 0.027; Close, *t*(11) = 0.49, Cohen's *d* = 0.08 [95% CI: −0.45, 0.62], *p* = 0.636; cf. Figure 3B,C). However, there were no significant differences in performance between stimulus sets seen in the Sleep and Wake conditions (no main effect of Sleep/Wake, *F*(1, 11) = 0.00, $\eta_{p}^{2}$ = 0.00, *p* = 1.00; no Sleep/wake × Prototype/Old interaction, *F*(1, 11) = 0.33, $\eta_{p}^{2}$ = 0.03, *p* = 0.577; no Sleep/Wake × Prototype/Old × Close/Distant interaction, *F*(1, 11) = 0.01, $\eta_{p}^{2}$ = 0.00, *p* = 0.927).

In summary, with regard to the initial recognition tests, we did not find main or interaction effects involving the Prototype/Old factor after 20 min and after 1 week. Conversely, 2‐AFC performance after 1 year indicates stronger memory for prototypes than for originally encoded items. This effect appears somewhat more pronounced for dissimilar items. Overall, however, gist representations do not seem to strongly depend on stimulus similarity during encoding. Moreover, the emergence and stability of gist memory did not differ between sleep and wake conditions across the first week after encoding.

### 3.2 Changes in Task Demands Explain Absence of Sleep Effects

The absence of sleep effects here contrast with our previous study (Lutz et al. 2017). Given that we started out with a sample of comparable size to Lutz et al. (2017), but obtained data from only 75% of participants during the final test after 1 year, we first assessed achieved power post‐hoc. Specifically, we tested whether a difference in prototype recognition between sleep and wake conditions after 1 year would have been detectable in our effective sample if it were of similar effect size as in our previous study, disregarding any hypothesized interactions. For a one‐tailed comparison with N=12, p=0.05, and 1−β=0.8, the required effect size is d=0.77. This is comparable to the effect observed in our previous study (d=0.56). Thus, the divergent results may be due to a lack of statistical power, in addition to differences in experimental protocols.

The main difference between the two experiments was the addition of a stimulus‐similarity factor in the present study. This was implemented via an increase of stimulus sets (40 sets per condition, compared with 16 in Lutz et al. 2017) and by splitting them into similar and dissimilar groups (resulting in 5 instead of 10 stimuli per set). We reasoned that encoding half as many stimuli per set for more than twice as many sets would increase task difficulty, and thus presented each set twice in a row.

**FIGURE 4:**
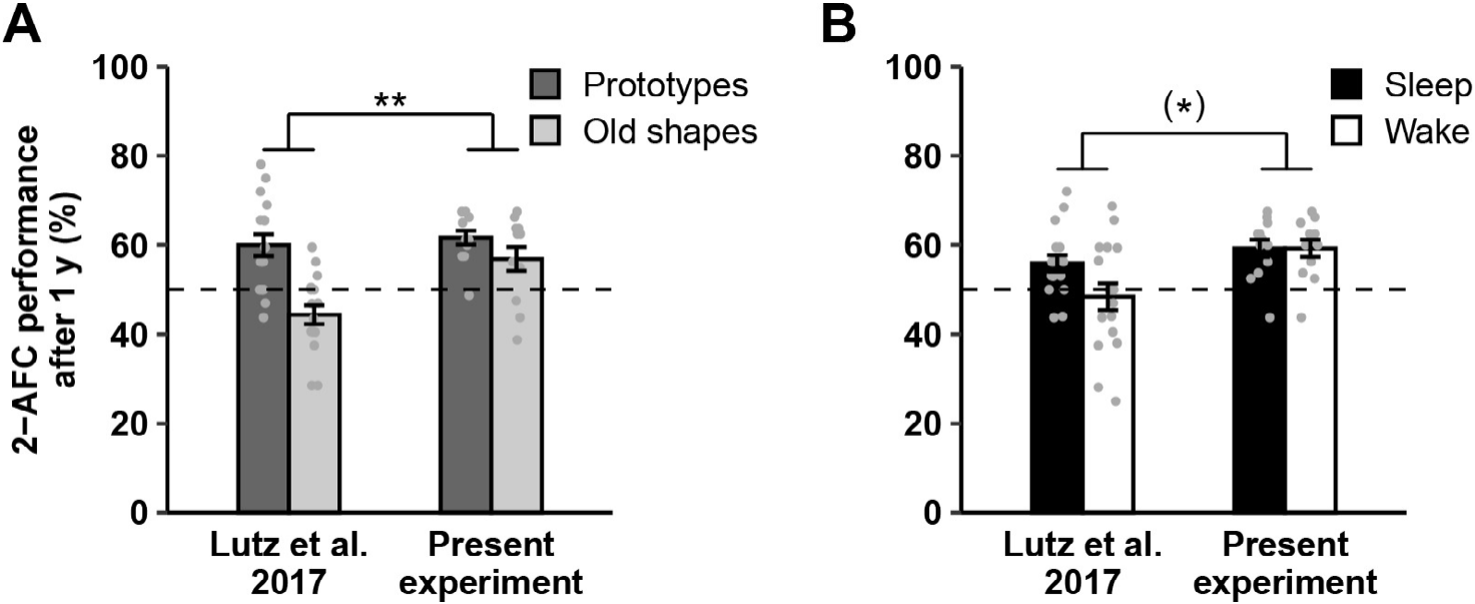
Comparison between (Lutz et al. 2017) and the present experiment at the one‐year test. Means ± SEM and individual data points are shown for 2‐AFC performance after 1 year across both experiments. (A) Performance for Prototypes and Old shapes differed in Lutz et al. (2017), but not in the present experiment (**p < 0.01 for the interaction between Experiment and Prototype/Old). (B) The effect of sleep vs. wakefulness on overall recognition performance tended to be larger in Lutz et al. (2017) than in the present experiment (*p < 0.1 for the interaction between Experiment and Sleep/Wake). *N* = 17 and *N* = 12 for the previous and present experiments, respectively.

To test whether these changes affected participants' performance, we collapsed the present data across stimulus similarity (which exerted only marginal effects, see above) and performed analyses across experiments at the 20‐min and one‐year tests. At the 20‐min test, we observed differential performance levels for prototypes and old shapes between the two experiments (interaction Prototype/Old × Experiment, *F*(1, 30) = 4.66, $\eta_{p}^{2}$ = 0.13, *p* = 0.039). This was driven by lower recognition levels for prototypes in the present experiment compared with Lutz et al. (2017). Conversely, at the one‐year test, we found higher overall performance in the present experiment (*F*(1, 27) = 7.11, $\eta_{p}^{2}$ = 0.21, *p* = 0.013). This main effect was mostly due to a larger difference in performance between prototypes and old stimuli in our previous experiment (interaction Prototype/Old × Experiment, *F*(1, 27) = 7.84, $\eta_{p}^{2}$ = 0.23, *p* = 0.009; Figure 4A). In addition, the effect of sleep vs. wakefulness on overall recognition performance tended to be larger in our previous experiment (interaction Sleep/Wake × Experiment, *F*(1, 27) = 3.18, $\eta_{p}^{2}$ = 0.11, *p* = 0.086; Figure 4B). These results may be explained by changes in experimental parameters such as the reduced number of items per set, the reduced similarity between items and prototypes for the distant sets, and viewing items twice during encoding, which likely rendered item and gist memories less distinct here than in our original study and may thereby have reduced the effect of sleep on gist abstraction.

## 4 Discussion

We investigated the effect of post‐encoding sleep on gist abstraction from visual shapes across retention intervals of 20 min, 1 week, and 1 year. We found that gist memory for prototypes not seen during initial encoding was evident early after encoding, persisted across 1 week, and exceeded veridical item memory after 1 year. Specifically, whereas gist and veridical memory performance were roughly comparable after 20 min and virtually identical after 1 week, we found better memory for prototypes compared with initially encoded items after 1 year. This effect was independent of whether participants slept or stayed awake after encoding, as we found no beneficial effect of sleep on gist abstraction across 1 week or 1 year of retention. We also did not find a beneficial effect of sleep on consolidation of veridical memories. Notably, overall memory tested after 1 week was even better after post‐encoding wakefulness than sleep. Taken together, our findings indicate that gist information is rapidly extracted and supports memory performance after 1 year of encoding, and that both its extraction and consolidation can occur independently of whether people sleep or stay awake immediately after encoding.

The difference between recognition of prototypes and old shapes after 1 year of retention is in line with our previous results (Lutz et al. 2017), as well as with current theories of memory transformation (Dudai et al. 2015; Nadel et al. 2012; Squire et al. 2015; Winocur and Moscovitch 2011) proposing that gist abstraction is a process that evolves slowly over time. Assuming that prototypes represent an overlap of multiple individual representations of old shapes, prototypes should be more strongly represented than old shapes and thus less prone to forgetting over time. This assumption is in line with the long‐held view of gist being an evolutionary adaptation to efficiently cope with the limited capacity of our brains to store information (e.g., Feld et al. 2016; Feld and Born 2017; Lewis and Durrant 2011; Lutz and Born 2019), and it agrees with earlier literature showing that gist memories are persistent and exceed recall of the originally encoded items after a delay of days (McDermott 1996; Neuschatz et al. 2001; D. G. Payne et al. 1996; Thapar and McDermott 2001), weeks (Posner and Keele 1970; Strange et al. 1970; Toglia et al. 1999) or months (Seamon et al. 2002; Zeng et al. 2021) following encoding. However, we also replicated our previous findings of high prototype recognition early after encoding (Lutz et al. 2017), suggesting that a substantial amount of gist information is extracted during or immediately after encoding. While this result is also in line with the previous work showing substantial levels of gist recall/recognition immediately after encoding (including short‐term memory tasks, e.g., Coane et al. 2007), additional studies are required to test whether gist representations during later measurements reflect persistence of such early memory traces, or whether these undergo additional qualitative changes in accordance with the notion of an active process of abstraction developing over extended periods of time. To conclusively answer this question will require tasks in which the same testing procedure can be used across long intervals. Here, we resorted to using a 2‐AFC procedure after 1 year to counter expected effects of memory decay, which in turn did not allow us to assess the stability of different memory traces in the same statistical model. In addition, future studies will need to resolve the issue of repeated presentation of prototype shapes before testing after prolonged time intervals. With the present protocol, gist abstraction can only be addressed during the 20‐min and one‐week intervals, that is, when the prototypes were not seen by the participants, whereas performance at the one‐year test cannot be used to distinguish gist abstraction from retention of prototypes once they have been presented during the immediate or 1‐week tests.

The present results contrast with our previous findings (Lutz et al. 2017) in that we did not observe a beneficial effect of sleep soon after encoding on gist memory across extended intervals of up to 1 year. On the one hand, this could be due to the true effect of sleep on gist abstraction being smaller than suggested by our original findings. If so, our decision to measure the same number of participants as in our previous experiment may have entailed a lack of statistical power. Alternatively, or additionally, changes to our original experimental protocol may have diminished the effect of sleep on gist abstraction. Two such changes in particular may help explain the divergent results: First, to obtain sufficient trial numbers despite the inclusion of additional experimental factors, we used a larger number of stimulus sets. Secondly, participants saw smaller sub‐sets of stimuli (five instead of 10 items) twice in a row (instead of only once) during encoding.

Direct comparisons across datasets showed lower recognition of prototypes shortly after encoding, but higher overall performance and reduced distinctiveness of prototype and item memory traces after 1 year in the present experiment. This may be linked to the reduced number of stimuli per set in the present experiment. While a meta‐analysis of verbal DRM studies found that shorter lists lead to increased sleep effects on false (i.e., gist) memory, only lists between 15 and 20 words long were used in studies analyzed (Newbury and Monaghan 2019). More recently, Mak et al. (2023) reported similar results for DRM lists consisting of eight words. Using even shorter lists of three to seven words, Coane et al. (2007) reported more false memories for longer lists when recognition was assessed directly after encoding. While caution is warranted when comparing verbal and non‐verbal protocols, these data suggest that our stimulus sets were at the lower limit for testing sleep effects on gist abstraction. Potential floor effects due to the small stimulus sets may have been compounded by the repeated presentation of stimuli, emphasising similarities and differences between individual stimuli and thereby supporting the emergence of stronger and more distinct representations of both individual items and their prototypes (Benjamin 2001; Bowman and Zeithamova 2020). Given evidence that sleep particularly reinforces memory traces of weak‐to‐intermediate strength, representations in the present experiment may have been sufficiently robust for sleep to only play a subordinate role in their consolidation, or to render consolidation processes during wakefulness equally effective (Bäuml et al. 2014; Cairney et al. 2016; Creery et al. 2015; Drosopoulos et al. 2007; Lo, Dijk, et al. 2014; Lutz et al. 2024; J. D. Payne et al. 2012; Petzka et al. 2021; Stickgold 2009). Indeed, item‐specific processing has been shown to diminish reliance on gist representations compared to relational processing, particularly in recognition tasks (Huff and Bodner 2013, 2018). To explore this possibility, we performed correlation analyses based on PSG data from the Sleep condition. Results indicated a link between long‐term gist abstraction and SOs as well as SO‐spindle co‐occurrence (Figure S4). Future studies should address the functional importance of these correlations by recording PSG data during delayed sleep in the Wake condition.

We also tested the hypothesis that stimuli sharing more features with their prototype would yield higher initial prototype recognition rates, and that sleep would be particularly important for extracting gist information from stimulus sets distant from their prototype. However, behavioural results showed no difference in recognition rates either 20 min or 1 week after encoding. And although we observed a marginally significant advantage for distant stimulus sets after 1 year, this effect was independent of the sleep/wake condition. Thus, feature overlap between stimuli and prototypes did not interact with consolidation during sleep or wakefulness to determine behavioural outcome.

## 5 Conclusion

In summary, our findings indicate that gist abstraction occurs soon after encoding visual shapes and leads to stable memories for non‐learned prototypes across intervals of at least 1 year. In contrast to a previous experiment based on a similar protocol, this behavioural outcome was independent of sleep soon after encoding. This divergence was likely linked to the repeated presentation of a limited number of stimuli during encoding and highlights the central role of task demands during encoding on the temporal evolution of memory consolidation and abstraction.

## Author Contributions

**Nicolas D. Lutz:** conceptualization, data curation, formal analysis, investigation, methodology, project administration, resources, software, supervision, visualization, writing – original draft, writing – review and editing. **Johanna Himbert:** investigation. **Jessica Palmieri:** conceptualization, investigation, methodology. **Eva‐Maria Kurz:** formal analysis, resources, software, visualization, writing – review and editing. **Isabel Raposo:** conceptualization, investigation. **Xuefeng Yang:** formal analysis. **Jan Born:** conceptualization, funding acquisition, methodology, supervision, validation, writing – review and editing. **Karsten Rauss:** conceptualization, formal analysis, funding acquisition, methodology, project administration, resources, software, supervision, validation, visualization, writing – original draft, writing – review and editing.

## Conflicts of Interest

The authors declare no conflicts of interest.

## Supporting information


**Data S1.** Supporting Information.

## Data Availability

Preprocessed data supporting the conclusions of the present study are available on the Open Science Framework: https://doi.org/10.17605/OSF.IO/J7A3R.
