# Supplementary material for: Long‐Term Visual Gist Abstraction Independent of Post‐Encoding Sleep
Source: J Sleep Res. 2025 Jun 9;35(1):e70106. doi: 10.1111/jsr.70106 (PMC12856105; doi:10.1111/jsr.70106)
Supplement: Supplementary file 1 — Data S1. Supporting Information. [file JSR-35-e70106-s001.pdf]

Long-term visual gist abstraction independent of post-encoding sleep: Supporting Information

Nicolas D. Lutz[1,2*], Johanna Himbert[1], Jessica Palmieri[1‡], Eva-Maria Kurz[1,3], Isabel Raposo[1,4‡‡], Xuefeng Yang[1,4], Jan Born[1,5,6,7], and Karsten Rauss[1*]

[1]Institute of Medical Psychology and Behavioral Neurobiology, University of Tübingen, Germany
[2]Institute of Medical Psychology, Faculty of Medicine, LMU Munich, Germany
[3]Department of Child and Adolescent Psychiatry, Psychosomatics, and Psychotherapy, University Hospital of Psychiatry and Psychotherapy, Tübingen, Germany
[4]Graduate Training Centre of Neuroscience/IMPRS for Cognitive & Systems Neuroscience, University of Tübingen, Germany
[5]Werner Reichardt Centre for Integrative Neuroscience, University of Tübingen, Germany
[6]German Center for Diabetes Research (DZD), Institute for Diabetes Research & Metabolic Diseases of the Helmholtz Center Munich at the University Tübingen (IDM), Germany
[7]German Center for Mental Health (DZPG) Tübingen, Germany

*Corresponding authors:
Nicolas D. Lutz, Institute of Medical Psychology, Faculty of Medicine, LMU Munich, Goethestr. 31, 80336 Munich, Germany; nicolas.lutz@med.uni-muenchen.de
Karsten Rauss, Institute of Medical Psychology and Behavioral Neurobiology
Eberhard Karls University Tübingen, Otfried-Müller-Str. 25, 72076 Tübingen, Germany; karsten.rauss@uni-tuebingen.de
‡Present address: Department of Psychology, University of Freiburg, 79085 Freiburg, Germany
‡‡Present address: Hertie Institute for Clinical Brain Research, University of Tübingen, 72076 Tübingen

Polysomnography and actigraphy measures

Participants showed normal sleep patterns during the experimental night as measured by polysomnography (**Table S1**). In addition to objective measures, participants filled a questionnaire on subjective sleep quality (SF-A/R; Görtelmeyer, 2011). On a scale from 1 (very little) to 5 (very high), overall sleep quality (SQ, 3.47 ± 0.21), feeling recovered after sleep (GES, 3.25 ± 0.18), mental balance before sleep (PSYA, 3.75 ± 0.15), mental exhaustion before sleep (PSYE, 2.88 ± 0.21), and psychosomatic symptoms during sleep (PSS, 1.27 ± 0.09) were all within normal range. Additionally, participants' general sleep quality (measured via the Pittsburgh Sleep Quality Index, PSQI) and chronotype (measured via the Morningness-Eveningness Questionnaire, MEQ; Horne & Ostberg, 1976) were assessed. All participants had a PSQI score ≤ 6 (3.63 ± 0.29 and 4.13 ± 0.29 in the Sleep and Wake conditions, respectively; $t(15) = 1.58$, Cohen's $d = -0.40$ [95% CI: -0.90, 0.12], $p = .135$). Participants were mostly intermediate chronotypes (2.94 ± 0.11, on a scale between 1 [definite evening type] and 5 [definite morning type]).

Table S1. Sleep parameters during the experimental night.

Sleep parameter	min	%	µV
Total sleep time	454.47 ± 2.98	-	-
Sleep onset latency	23.67 ± 3.23	-	-
S1	51.53 ± 7.79	11.44 ± 1.57	-
S2	226.60 ± 9.55	49.89 ± 2.08	-
SWS	64.57 ± 4.55	14.20 ± 0.97	-
REM sleep	74.10 ± 5.48	16.29 ± 1.17	-
WASO	19.10 ± 7.55	4.21 ± 1.67	-
Sleep efficiency	-	91.05 ± 1.67	-
Fast spindle amplitude	-	-	20.41 ± 1.39
SO amplitude	-	-	169.85 ± 6.43
Co-occurrence of spindles with SO	-	14.74 ± 1.14	-

Mean ± SEM are shown. SWS, slow-wave sleep; REM, rapid eye-movement sleep; WASO, wake after sleep onset; SO, slow oscillations. Percentages of S1, S2, SWS, REM sleep and WASO were calculated as percent of total sleep time. Sleep efficiency was calculated as total sleep time divided by the sleep period (i.e., the period from sleep onset to waking up). Co-occurrence of spindles with SO was calculated as percentage of spindles coinciding with a SO (SO trough) ± 1.2 s around the spindle trough. N = 15 for classic sleep parameters, N = 14 for fast spindle amplitude, SO amplitude, and co-occurrence of spindles with SO.

To assess if participants had normal sleep/wake cycles during the seven nights between encoding and the one-week test, we collected actigraphy data and had the participants keep sleep journals. There was no significant difference between Sleep and Wake conditions for the parameters interdaily stability (i.e., the stability of rest-activity rhythms between different days), intradaily variability (i.e., the fragmentation of a rest-activity pattern), or the relative amplitude between the 10 hours of maximum activity and the five hours of lowest activity ($p \geq .239$), based on non-parametric circadian rhythm analysis (NPCRA; Blume et al., 2016). In addition, we did not find any difference between Sleep

and Wake conditions for sleep parameters during the last night before the one-week test (p ≥ .186; **Table S2**).

Table S2. Actigraphy

	Sleep condition	Wake condition
Seven days between encoding and one-week test		
Interdaily stability	0.47 ± 0.04	0.47 ± 0.03
Intradaily variability	0.94 ± 0.06	1.00 ± 0.09
Relative amplitude	0.93 ± 0.01	0.93 ± 0.01
Night before one-week test		
Time in bed (min)	497 ± 16.5	520 ± 11.2
Total sleep time (min)	392 ± 13.5	397 ± 13.9
WASO (%)	17.8 ± 1.22	17.7 ± 0.94
Sleep efficiency (%)	79.0 ± 1.27	76.2 ± 1.81

Mean ± SEM are shown. Interdaily stability, intradaily variability, and relative amplitude are measures derived from non-parametric circadian rhythm analyses (NPCRA). These measures, as well as measures for time in bed, total sleep time, wake after sleep onset (WASO), and sleep efficiency were calculated using a standard algorithm as implemented in MotionWare 1.2.5 (RRID:SCR_022253), with a high sensitivity threshold for the detection of wake phases and an epoch length of 15 s. N = 16.

Prototypicality tests

To investigate whether prototypes in our task were in fact perceived by the participants as prototypical for the shapes of a set, as previously assumed (Diekelmann et al., 2011; Lutz et al., 2017), we further took several control measures in the present study. First, we directly compared participants' recognition of prototypes with related shapes (both of which have not been seen during previous encoding) in the recognition test. Prototypes were recognized significantly more often as 'old' than related shapes ($F(1,15) = 22.77$, $\eta_p^2 = 0.60$, $p < .001$; **Figure S1**), suggesting that prototypes are qualitatively different from other shapes of the same set that had also not been seen previously. This difference did not depend on whether related shapes were from distant or close subsets (no Close/Distant x Prototype/Related interaction, $F(1,15) = 0.49$, $\eta_p^2 = 0.03$, $p = .495$), and there was no evidence that recognition rates for related shapes differed between close and distant subsets ($F(1,15) = 0.09$, $\eta_p^2 = 0.006$, $p = .770$).

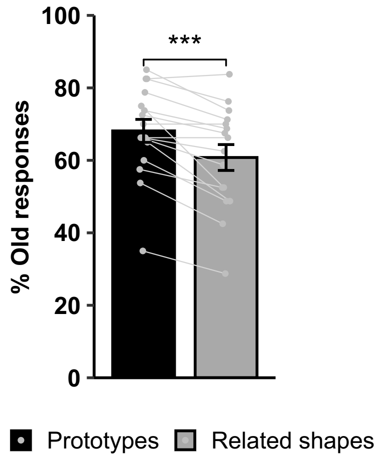

Figure S1. Recognition of prototypes and related shapes. *Means ± SEM and individual data points are shown for the recall performance of prototypes and related shapes, averaged across Sleep and Wake conditions as well as across the 20-min and one-week tests. ***p<0.001. N = 16.*

In addition, we invited participants to a separate session after the one-week test of their second condition to perform a set rating task, in which they were asked to choose two shapes of each set that appeared most representative of the whole set (i.e., that are prototypical; in descending order). Compared to chance level (i.e., 1 out of 11 = 9.10% for prototypes and 5 out of 11 = 45.45% for old and related shapes, respectively), participants' selection of the actual prototypes was significantly above chance level ($t(14) = 4.18$, Cohen's d = 1.08 [95% CI: 0.43, 1.71], $p < .001$), whereas old shapes or related shapes were selected at chance ($t(14) = 0.69$, Cohen's d = -0.18 [95% CI: -0.69, 0.34], $p = .503$) or below chance level ($t(14) = 4.02$, Cohen's d = -1.04 [95% CI: -1.66, -0.39], $p = .001$), respectively (**Figure S2**). This pattern was independent of whether participants had slept or stayed awake after encoding of the respective sets (no main effect of Sleep/wake, $F(2,14) = 3.24^{-14}$, $\eta_p^2 = 0.00$, $p = 1.000$; no Sleep/wake x Stimulus type interaction, $F(2,28) = 0.51$, $\eta_p^2 = 0.04$, $p = .552$).

Similarly, when considering the participants' two choices together, only participants' selection of the actual prototypes was significantly above chance level ($t(14) = 4.11$, Cohen's d = 1.06 [95% CI:

0.41, 1.69], p = .001; chance level: $\left(\frac{1}{11} * \frac{10}{10}\right) + \left(\frac{10}{11} * \frac{1}{10}\right) = 18.19\%$), whereas both old and related shapes were selected significantly below chance level (t(14) = 18.83, Cohen's d = -4.86 [95% CI: -6.70, -3.00], p < .001 and t(14) = 11.40, Cohen's d = -2.94 [95% CI: -4.12, -1.74], p < .001, respectively; chance level: $\left(\frac{5}{11} * \frac{4}{10}\right) + \left(\frac{5}{11} * \frac{6}{10}\right) + \left(\frac{6}{11} * \frac{5}{10}\right) = 72.72\%$). Again, there was no difference between sleep or wakefulness (no main effect of Sleep/wake, F(2,14) = 0.50, η_p^2 = 0.04, p = .490; no Sleep/wake x Stimulus type interaction, F(2,28) = 0.37, η_p^2 = 0.03, p = .606).

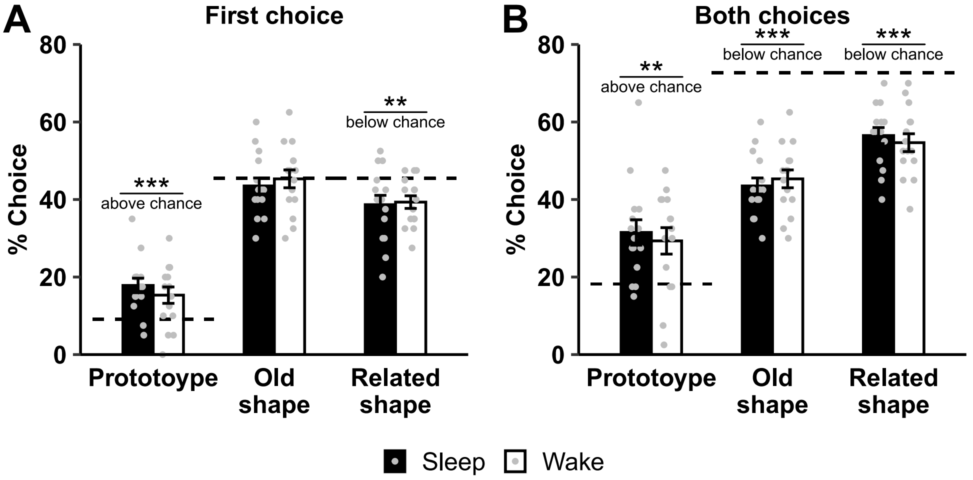

Figure S2. Set rating. *Means ± SEM and individual data points are shown for the first choice (**A**) or both choices of either prototypes, old shapes, or related shapes (**B**) in the set rating task (across both Sleep and Wake conditions). ***p<0.001, **p<0.01. N = 15.*

Tests of general memory performance

To investigate whether participants actually encoded the presented visual shapes, we compared recognition rates for encoded sets of shapes with new shapes. An ANOVA including all four stimulus types (Prototype/Old shapes/Related shapes/New shapes) yielded a significant main effect of Stimulus type ($F(1,45) = 33.84$, $\eta_p^2 = 0.69$, $p < .001$; **Figure S3**), and individual comparisons showed significantly lower recognition rates for new shapes vs. prototypes ($t(15) = 7.61$, Cohen's d = 0.76 [95% CI: 0.26, 1.27], $p < .001$), old shapes ($t(15) = 7.12$, Cohen's d = 0.87 [95% CI: 0.31, 1.42], $p < .001$), and related shapes ($t(15) = 3.70$, Cohen's d = 0.31 [95% CI: -0.03, 0.64], $p = .002$). These results suggest that participants were able to differentiate encoded and non-encoded "new" sets of shapes in the present study.

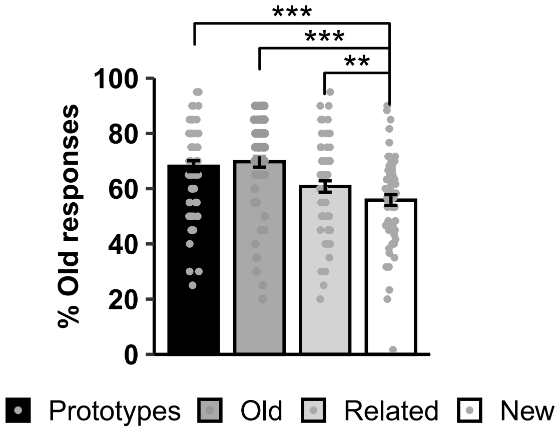

Figure S3. Recognition of all stimulus types. *Means ± SEM and individual data points are shown for the recall performance of prototypes, old, related, and new shapes, averaged across Sleep and Wake conditions as well as across the 20-min and one-week tests. ***p<0.001, **p=0.002. N = 16.*

Remember/know/guess judgements and confidence ratings

In addition to testing participants' recognition performance, we also asked them to indicate if they explicitly remembered a presented shape, if they knew it (i.e., if they had a feeling of familiarity without being able to remember specific contextual details), or if they had to guess. They were also asked to rate their confidence on a 4-point scale. We did not find any difference between Sleep and Wake conditions at either 20-min or one-week tests for either remember/know/guess judgements or confidence ratings (individual paired t-tests, all p ≥ .125; **Table S3**).

Table S3. Remember/know/guess judgements and confidence ratings during the 20-min and one-week tests in both Sleep and Wake conditions.

		20-min test		One-week test	
		Sleep	Wake	Sleep	Wake
Remember	Prototypes	0.14 ± 0.03	0.15 ± 0.04	0.04 ± 0.02	0.07 ± 0.02
	Old shapes	0.17 ± 0.03	0.21 ± 0.04	0.06 ± 0.02	0.09 ± 0.02
Know	Prototypes	0.52 ± 0.04	0.48 ± 0.05	0.50 ± 0.04	0.44 ± 0.04
	Old shapes	0.46 ± 0.05	0.39 ± 0.03	0.51 ± 0.02	0.49 ± 0.04
Guess	Prototypes	0.34 ± 0.04	0.38 ± 0.04	0.46 ± 0.05	0.48 ± 0.05
	Old shapes	0.38 ± 0.04	0.41 ± 0.04	0.43 ± 0.03	0.42 ± 0.04
Confidence	Prototypes	2.40 ± 0.09	2.37 ± 0.09	2.19 ± 0.09	2.21 ± 0.10
	Old shapes	2.34 ± 0.10	2.39 ± 0.09	2.15 ± 0.08	2.21 ± 0.09

Note: Mean ± SEM are shown (remember/know/guess in %, confidence 1 – 4)

Control tests

During the adaptation-night session, participants' word fluency (Aschenbrenner et al., 2000) was tested. In addition, we measured participants' vigilance (Diekelmann et al., 2013) as well as their subjective sleepiness (Stanford Sleepiness Scale, SSS; Hoddes et al., 1972) during the encoding session, the one-week test session and the set rating session. Finally, we also tested participants' digit span during the encoding and the one-week test sessions. There were no significant differences between Sleep and Wake conditions on any of these measures (all $p \geq .216$; **Table S4**).

Table S4. Vigilance, sleepiness, and digit span

	Sleep condition	Wake condition
Adaptation night		
Word fluency (# words)	16.70 ± 1.36	
Encoding		
Vigilance (ms)	414 ± 8.42	419 ± 10.50
Sleepiness	3.63 ± 0.13	3.44 ± 0.16
Digit span	6.94 ± 0.27	6.94 ± 0.30
One-week test		
Vigilance (ms)	410 ± 9.96	411 ± 12.10
Sleepiness	2.50 ± 0.24	2.25 ± 0.19
Digit span	7.19 ± 0.28	6.94 ± 0.36
Set rating		
Vigilance (ms)	406 ± 9.58	
Sleepiness	2.27 ± 0.24	

Mean ± SEM are shown. Word fluency was tested with the German Regensburger Wortflüssigkeitstest. Participants were asked to write down as many words as possible starting with a specific letter (letter "p") during a 2-min interval. Vigilance was measured using a Psychomotor Vigilance Task. Participants were asked to respond as fast and correctly as possible to a dot that was shown either on the left or the right side of the screen. Sleepiness was measured using the Stanford Sleepiness Scale (7-point-scale on sleepiness). Digit span was measured in the forward direction with a starting length of three digits, two repetitions per sequence length, and a presentation duration of 1 s. N = 16.

Correlations between sleep parameters and behavioral performance

We used PSG recordings obtained in the Sleep condition to assess sleep parameters known to be involved in memory reprocessing, i.e., sleep spindles, SOs, and their co-occurrence (Klinzing et al., 2019). Given the purely visual nature of our task, we reasoned that any association between sleep microstructure and behavioral performance should be most pronounced at parieto-occipital leads (cf. Lutz et al., 2021). We thus limited our analyses to electrodes P3, P4, O1, and O2 and investigated only fast spindles, as they usually show a posterior focus. An exception was made for SO-spindle co-occurrence, which was calculated across all electrodes because SOs usually show a frontal distribution, and we did not want to artificially reduce the number of co-occurrences given the distinct topographical distributions of the underlying events.

Performance during the one-week test did not correlate with any fast spindle parameters (all $p \geq .085$). In contrast, all SO parameters (i.e., count, density, amplitude, and power density) were associated with higher performance for prototypes from distant subsets at occipital electrodes only (all $p \leq .034$). This finding was specific in that the only other significant correlations were between SO amplitude and veridical memory for both close and distant sets at both parietal and occipital electrodes (all $p \leq .042$). Finally, co-occurrence of SOs and fast spindles was not correlated with raw performance measures (all $p \geq .22$). However, it predicted the difference in performance between prototypes and old items from distant subsets ($r = 0.572$, $p = .033$), a compound measure based on memory transformation theory (Winocur & Moscovitch, 2011; Yassa & Reagh, 2013) that operationalizes gist abstraction as a process that benefits recognition of prototypes while detailed memory of old shapes is increasingly forgotten (**Figure S4 A-D**).

For the one-year test, there were no associations between 2-AFC performance and fast spindles (all $p \geq .079$). Correlations for distant prototypes at occipital electrodes remained significant only for SO amplitude ($r = 0.593$, $p = .042$) and power density ($r = 0.581$, $p = .048$) (**Figure S4 E-F**). Paradoxically, SO-spindle co-occurrence was positively correlated with performance for prototypes from close ($r = 0.577$, $p = .049$) but not distant subsets ($r = -0.235$, $p = .462$). This highlights the fact that the uncorrected correlations reported above need to be interpreted with caution. Nevertheless, the consistent and topographically specific results for SOs is in line with the view that memory reprocessing during sleep soon after encoding supports visual gist abstraction.

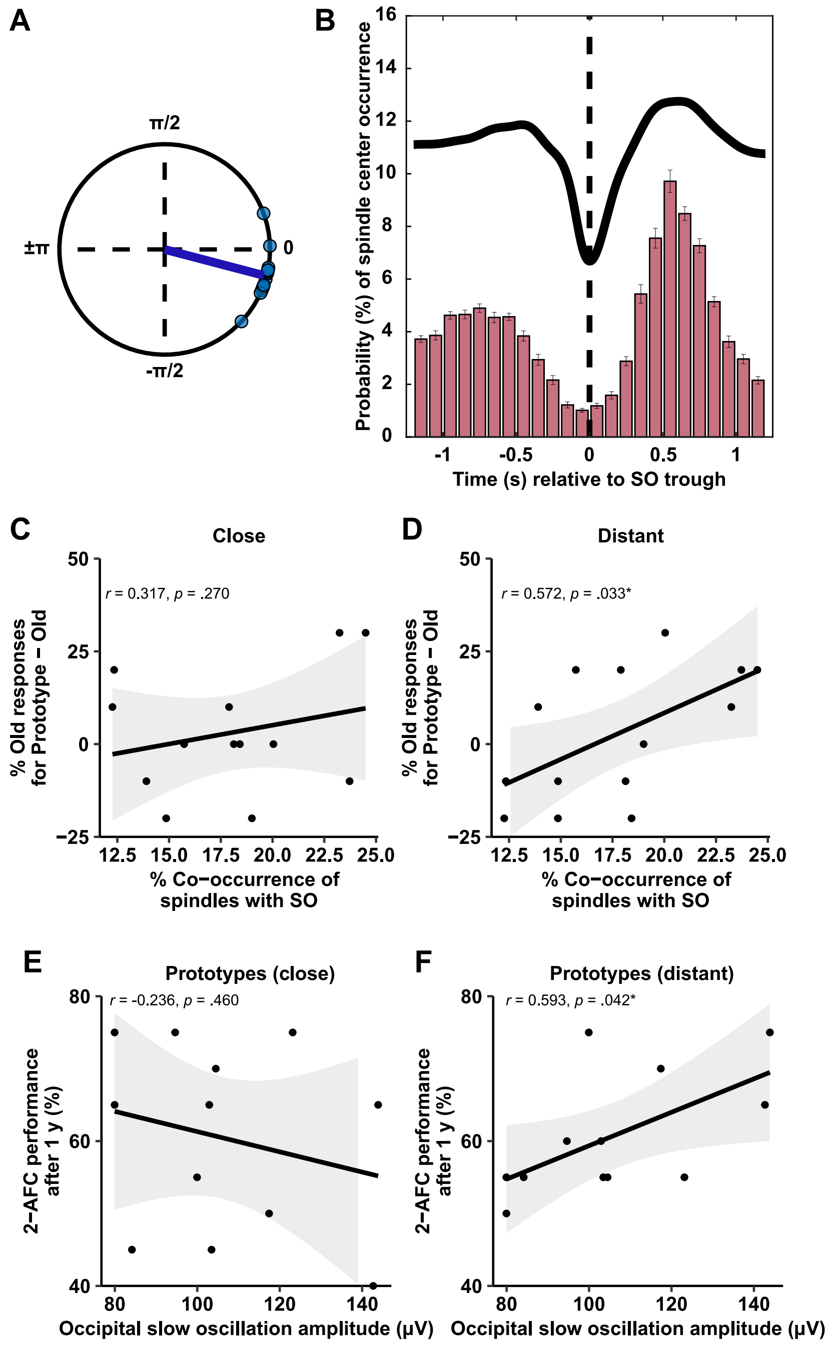

Figure S4*. *Correlations between behavioral performance, SO-spindle co-occurrence, and SO amplitude at retrievals after one week and one year. *(**A**) Mean preferred phase (blue dots) and coupling strength (blue line) for the coupling of fast spindles to the SO cycle (0°, SO-up-state peak; ±180°; SO-down-state peak) averaged across frontal, central, parietal, and occipital electrode sites. (**B**) Peri-event time histograms depicting the rate of fast spindle occurrence within 100-ms bins ±1.2-s around the SO down-state peak (0 s, vertical dashed line) at averaged frontal, central, parietal, and occipital electrode sites. (**C**-**D**) Scatter plots including 95% confidence intervals (shading) for correlations between the difference of % old responses between prototypes and old shapes and the % co-occurrence of fast spindles with SO for close (**C**) and distant (**D**) subsets at the one-week test. (**E**-**F**) Scatter plots for correlations between occipital SO amplitude and two-alternative forced-choice (2-AFC) performance for prototypes in close (**E**) and distant (**F**) subsets at the one-year test. N = 14 and N = 12 for one-week and one-year tests, respectively.*
